# A comparison of transhepatic versus transperitoneal cholecystostomy for acute calculous cholecystitis: a 5-year experience

**DOI:** 10.1093/jscr/rjab410

**Published:** 2021-09-14

**Authors:** Stephen Bennett, Nadeem Shaida, Edmund Godfrey, Peter Safranek, J Robert O’Neill

**Affiliations:** Cambridge Oesophago-Gastric Centre, Cambridge University Hospitals NHS Foundation Trust, Cambridge CB2 0QQ, UK; Department of Radiology, Cambridge University Hospitals NHS Foundation Trust, Cambridge CB2 0QQ, UK; Department of Radiology, Cambridge University Hospitals NHS Foundation Trust, Cambridge CB2 0QQ, UK; Cambridge Oesophago-Gastric Centre, Cambridge University Hospitals NHS Foundation Trust, Cambridge CB2 0QQ, UK; Cambridge Oesophago-Gastric Centre, Cambridge University Hospitals NHS Foundation Trust, Cambridge CB2 0QQ, UK

## Abstract

Percutaneous cholecystostomy is a treatment for acute calculous cholecystitis used in patients where surgery is high risk or challenging either to allow for surgical optimisation or as definitive treatment. In this case series we compare the outcomes of a transhepatic versus transperitoneal approach in patients undergoing percutaneous cholecystostomy for acute calculous cholecystitis. A retrospective review of patients from 2014 to 2019 was conducted and included demographics, percutaneous cholecystostomy route, complications and outcome. Fifty-one patients were included. Percutaneous cholecystostomy was placed transhepatically in 15 cases; transperitoneal in 30 cases; 6 cases had undetermined route. The transhepatic cohort had 43.5% fewer readmissions due to biliary sepsis, 32.5% fewer drain-related complications, and were less likely to require further treatment (32.5% reduction) compared to the transperitoneal cohort. In our experience, the transhepatic route is preferred due to fewer complications, fewer readmissions and a reduction in the need for further treatment.

## INTRODUCTION

Acute cholecystitis is a frequent cause of emergency general surgical admission, with up to 95% of cases attributed to gallstone disease. Given increasing age is a strong risk factor for gallstone disease, and with an ageing population, acute calculous cholecystitis (ACC) will likely represent an increasing burden on Western healthcare systems for the foreseeable future [1].

Cholecystectomy is widely regarded as the best treatment for ACC in patients who are deemed fit for surgery. However, patients at high risk for surgery or in those whose protracted illness makes surgery challenging due to significant inflammation, cholecystectomy can lead to significant morbidity and mortality [2]. Percutaneous cholecystostomy (PC), either as a bridging measure to surgery or as a definitive treatment, is a valuable treatment option in this subset of patients. International guidelines recommend that it should be considered in patients with moderate or severe ACC, those with severe systemic disease, or patients who have failed medical therapy [3, 4].

Although surgical cholecystostomy was first published in 1867 [5], PC was first reported as a treatment for obstructive jaundice in 1979 [6] and later as a treatment for gallbladder empyema in 1980 [7]. PC is an image-guided, radiological procedure, directly decompressing the infected gallbladder contents, thereby reducing inflammation and improving patients’ general condition [8]. It has been shown to be safe in patients with multiple comorbidities, with a reported mortality rate of 12% and a complication rate of 4% in patients specifically with ACC [9]. PC can be placed via a transhepatic (TH) or transperitoneal (TP) route. With only limited series existing in the literature showing similar outcomes for both techniques, the optimal choice of PC route remains a much debated topic [10, 11].

We sought to compare the outcomes for patients undergoing PC for ACC via a TH or TP approach in a tertiary centre over a 5-year period. The outcome measures chosen included the need for further hospital admission due to biliary sepsis or drain-related complications, endoscopic retrograde cholangiopancreatography (ERCP), cholecystectomy and death due to biliary sepsis.

## CASE SERIES

### METHODS

Patients undergoing PC for ACC at a UK tertiary hospital between November 2014 and June 2019 were retrospectively identified. Patients were identified from a search of the hospital’s electronic health records database using the keywords ‘percutaneous drainage of gallbladder’, ‘cholecystostomy during an associated admission’ and ‘ultrasound guided drain of abdominal abscess’. To ensure inclusion of all patients, a search of the relevant current procedural terminology codes used at our hospital for PC placement was performed. Patient’s records were then reviewed to exclude any patients that underwent PC insertion for conditions other than ACC and to collect data on demographics, comorbidities, PC management, PC related complications, length of hospital stay and outcomes. Radiological reports and imaging were used to determine PC route.

PC insertion was performed using a Seldinger technique by a radiologist under local anaesthesia using either ultrasonography or computed tomography (CT) for image guidance. Access to the gallbladder was directed through a TH or TP route based on the preference of the radiologist and anatomy of the patient. The gallbladder was then aspirated to dryness and samples sent for microbiological and cytological analysis.

Patients were followed for up to 12 months. PC complications were included in the analysis if they required treatment or resulted in the presentation of the patient at the emergency department following discharge and included bile leak, infection of PC insertion site (wound infection), bleeding from PC insertion site (wound bleeding), PC insertion site pain, drain blockage, accidental drain dislodgement or removal, and chronic fistula. All bile leaks were confirmed by CT imaging. A drain was classed as being ‘blocked’ if aspiration was not possible and CT imaging confirmed a distended gallbladder, ‘dislodged’ if it remained in the patient but was confirmed on imaging as being outside of the gallbladder lumen.

A patient was classed as having no further treatment if they recovered from the initial attack of ACC to the point of discharge and did not require further hospital admission due to biliary sepsis or drain-related complications, ERCP or did not progress to cholecystectomy.

Statistical analysis was performed using IBM SPSS software version 26 (SPSS Inc., Chicago, IL, USA). Categorical variables were compared by the Fisher’s exact test. A two-tailed *P* value of ≤0.05 was considered statistically significant.

## RESULTS

A total of 78 patients underwent PC insertion at our centre between November 2014 and June 2019. Of these procedures, 52 were treatment for ACC, the remainder being for acalculous cholecystitis, malignancy and traumatic gallbladder perforation. PC was performed by 39 different radiologists/radiology trainees and placed via a TH route in 15 cases, TP route in 31 cases and for 6 cases it was not possible to determine the route retrospectively. Sixty-seven percent (31/46) of patients were male and the mean age at insertion was 73 (27–96) years (Table 1). The baseline patient characteristics were similar with the exception of the number of patients with cardiovascular disease in the TP group versus the TH group (65% and 11%, respectively).

**Table 1:** Patient characteristics

	Overall (*n* = 46)	Transhepatic (*n* = 15)	Transperitoneal (*n* = 31)
Mean age (years)	73 (27–96)	72 (40–86)	74 (27–96)
Male	31 (67%)	10 (67%)	21 (68%)
Female	15 (33%)	5 (33%)	10 (32%)
Comorbidities:			
Diabetes		6 (40%)	9 (29%)
Cardiovascular disease		8 (11%)	20 (65%)
Pulmonary disease		3 (20%)	11 (36%)
Chronic kidney disease		4 (27%)	6 (19%)
Immunosuppression		4 (27%)	4 (13%)
Premorbid function:			
ADL independent^a^		9 (60%)	21 (68%)
ADL assisted		4 (27%)	8 (26%)
Fully dependent		2 (13%)	2 (7%)

a ADL, activities of daily living.

One patient was excluded from the outcome analysis in the TP group because they underwent liver transplantation shortly after PC insertion (14 days) during the same admission due to deteriorating liver function.

Clinical outcomes are presented in Table 2. Follow-up ranged from 6 to 12 months with the median follow-up times for each group being 12 months. In summary, there were more PC related complications in the TP group compared to the TH group (40% versus 27%, *P* = 0.514) with 75% of these being due to the drain becoming dislodged or falling out. The TP group also had a greater proportion of patients being readmitted for biliary sepsis, 23% versus 13%, respectively (*P* = 0.695). However, a greater proportion of the TH group progressed to ERCP or cholecystectomy compared to the TP group, 33% versus 23% (*P* = 0.496) and 27% versus 20% (*P* = 0.710), respectively. Mortality due to complications of biliary sepsis was the same for both groups (7%). In the TH group, 40% of patients required no further treatment for their ACC following PC insertion, compared to 27% in the TP group (*P* = 0.497).

**Table 2:** Patient outcomes

	Overall (*n* = 45)	Transhepatic (*n* = 15)	Transperitoneal (*n* = 30)
Readmission for biliary sepsis	9 (18%)	2 (13%)	7 (23%)
Drain-related complications^a^:	16 (36%)	4 (27%)	12 (40%)
Bile leak	2 (4%)	0 (0%)	2 (7%)
Wound infection	1 (2%)	0 (0%)	1 (3%)
Wound bleeding	1 (2%)	1 (7%)	0 (0%)
Pain	1 (2%)	1 (7%)	0 (0%)
Drain blockage	1 (2%)	1 (7%)	0 (0%)
Drain dislodged	2 (4%)	0 (0%)	2 (7%)
Drain fell out	8 (18%)	1 (7%)	7 (23%)
Chronic fistula	1 (2%)	0 (0%)	1 (3%)
ERCP^b^	12 (27%)	5 (33%)	7 (23%)
Cholecystectomy:	10 (22%)	4 (27%)	6 (20%)
Elective	7 (16%)	3 (20%)	4 (13%)
Emergency	3 (6%)	1 (7%)	2 (7%)
Death due to complication of biliary sepsis	3 (6%)	1 (7%)	2 (7%)
No further treatment^c^	14 (31%)	6 (40%)	8 (27%)

a Complications were included if they required treatment or resulted in presentation at the emergency department following discharge.

b Endoscopic retrograde cholangiopancreatography.

c Further treatment classified as additional admission due to biliary sepsis, drain complication, ERCP or cholecystectomy.

There was little difference in length of hospital stay between the TH and TP groups, with the median being 20 days and 19 days, respectively. Considering PC management, the median time to cholangiogram following PC insertion was 6 days in the TH group and 9 days in the TP group. The median length of time the PC drain was left in place before purposeful removal in the TH group was 25 days compared to 34 days in the TP group.

## DISCUSSION

Historically TH placement has been favoured in the literature over TP despite a lack of comparative studies, the rationale for this being a decreased risk of bile leak, catheter dislodgement, and a decreased time to catheter track maturation [11]. The largest review to date, including 371 patients over a 13-year period, directly compared the two approaches in patients undergoing PC for any reason and reported no significant difference between TH and TP approaches in complication rates or outcomes [11]. It should be noted, however, that this Beland *et al.* study included patients undergoing PC for any reason, a clinically different population of patients compared to those included in our case series who were treated specifically for ACC. Only 10% of patients in the TH group and 10% of patients in the TP group experienced their catheter falling out or becoming dislodged in contrast to our findings of 7% and 30%, respectively. They also reported bile leakage rates of 4% (TH group) and 3% (TP group); in contrast to the 2 bile leaks seen in our review, both occurring in the TP group (7%). Overall, the complication rate was notably higher in the TP group compared to the TH group in our case series.

A prospective study by Hatjidakis *et al.* concluded that only 2 weeks are required to develop a mature tract when the TH access route is used compared to 3 weeks using the TP route [12]. In our case series, the median length of time the PC drain remained in situ was 25 days in the TH group compared to 34 days in the TP group.

The only randomized controlled trial comparing laparoscopic cholecystectomy with PC in patients with ACC was published in 2018 and reported that laparoscopic cholecystectomy was superior to PC in the treatment of high-risk ACC patients. The authors also concluded that PC was still an appropriate treatment in patients with a strict contraindication for surgery. Of the 68 patients included in the PC group, 41% required emergency readmission to hospital due to recurrent biliary disease [2]. This is a higher rate than that seen in our case series (18%) and interestingly we also observed a difference for this when comparing the two approaches (23% in the TP group versus 13% in the TH group). The CHOCOLATE trial also reported a higher rate of progression to surgery (46%) compared to our case series (22%), which may in part be due to the exclusion of critically ill patients with an ‘acute physiology and chronic health disease classification system II’ (APACHE II) score of ≥15 and those admitted to the intensive care unit [2]. Of note, we also observed a difference between approaches in the proportion of patients requiring no further treatment for their ACC following PC insertion, with a greater percentage being seen in the TH group (40% versus 27%), although the TH group was also more likely to progress to surgery (27% versus 20%). Our overall mortality rate of 16% is towards the lower end of the range (14%–30%) reported in the literature for laparoscopic cholecystectomy in elderly or critically ill patients with comorbid disease [4]. Our results, therefore, suggest that PC is a valuable treatment option for ACC, not only as a temporizing measure but, in selected cases, as definitive treatment.

The limitations of this case series include the relatively small sample size which likely impacted the achievement of statistical significance. It is also possible that a patient may have presented to another hospital for subsequent treatment, resulting in this not being captured in the data. However, this is unlikely given that all patients presented to our centre with their initial episode of ACC (suggesting they would return for subsequent treatment) and because all patients were followed up for 6–12 months, during which time they were seen in outpatient clinic and all relevant episodes recorded. Due to the retrospective design and the fact that PC route was based on the preference of the radiologist performing the procedure, it is possible that this could have resulted in selection bias. However, given that all patients undergoing PC for ACC at our tertiary centre over a 5-year period were included, the authors feel this represents a meaningful body of work in a subject area where PC route is much debated and with limited prospective trials.

We believe our case series to be one of the largest to date comparing the outcomes for patients undergoing PC via a TH or TP approach specifically for the treatment of ACC. In our experience, PC is an effective and relatively safe treatment for ACC in cases where surgery is high risk or challenging both to allow for surgical optimisation and as definitive treatment. In the absence of more prospective trials, we prefer using a TH route due to our observations of fewer complications, fewer readmissions due to biliary sepsis and a reduction in the need for further treatment compared to a TP route.

## CONFLICT OF INTEREST STATEMENT

None declared.
